# Study on MnO_2_/MXene–Ti_3_C_2_ composite materials as cathode materials for magnesium batteries

**DOI:** 10.1039/c9ra07652b

**Published:** 2019-10-18

**Authors:** Yuan Li, Donghui Xu, Dehang Zhang, Yuanchi Wei, Ruinan Zhang, Yuxiang Guo

**Affiliations:** Department of Materials & Metallurgy, University of Science and Technology Liaoning Anshan 114051 China gyxwsd@126.com

## Abstract

In this paper, MnO_2_/MXene–Ti_3_C_2_ composites with different molar ratios were successfully prepared by a one-step hydro-thermal method, and the optimum proportion was confirmed by XRD and SEM comparative analysis. The optimum proportion of MnO_2_/MXene–Ti_3_C_2_ composites and MnO_2_ was used as a cathode material for magnesium batteries to carry out the electrochemical performance test. The results showed that the charge–discharge capacity of the MnO_2_/MXene–Ti_3_C_2_ composite was up to 105 mA h g^−1^, much higher than that of MnO_2_ (64 mA h g^−1^), and meanwhile it had good rate performance. At the same time, this also opened up the application of MXene–Ti_3_C_2_, a new two-dimensional material, in the field of battery electrode materials.

## Abstract 2

In this paper, MnO_2_/MXene–Ti_3_C_2_ composites with different molar ratios were successfully prepared by a one-step hydro-thermal method, and the optimum proportion was confirmed by XRD and SEM comparative analysis.

## Graphical abstract

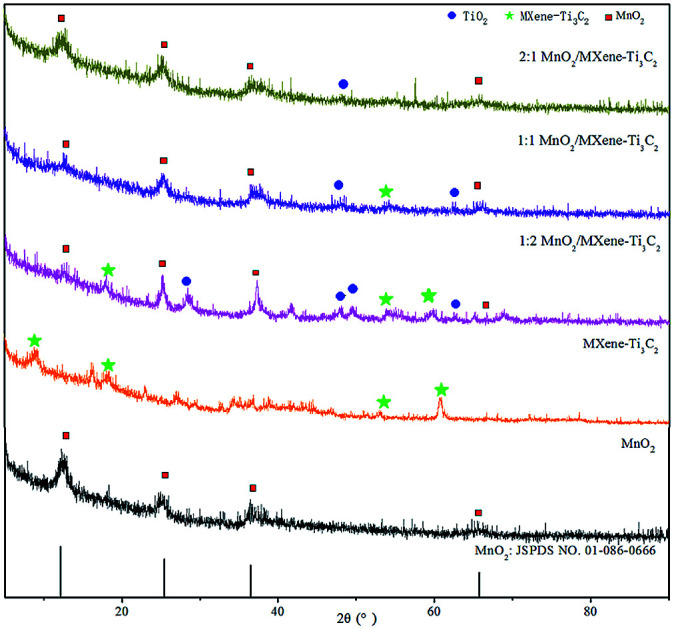

## 1. Introduction

With the rapid development of social economy, increasing consumption of fossil energy and escalating environmental pollution, it is extremely urgent to find clean, environmentally friendly, efficient and sustainable energy sources.^1–3^ Therefore, some new environmentally friendly secondary batteries with high-capacity have been favored by domestic and overseas researchers. Among them, lithium-ion batteries are widely used because of their high energy density and mature technology, but they have the problems of high cost and poor safety.^4,5^ Magnesium and lithium are located in a diagonal position in the periodic table, and have similar ionic radii and chemical properties. Compared with lithium metal (180.5 °C), the melting point of magnesium (648.8 °C) is much higher, and its activity is poorer. So, it is easy to process and also safer. Moreover, magnesium ions can carry two charges. Although the mass specific capacity (2205 mA h g^−1^) is not as high as lithium metal (3862 mA h g^−1^), it is still considerable.^6–9^ At the same time, China is rich in magnesium resources, ranking first in the world, and the price is low and it's easy to obtain.^10–12^ So, magnesium ion batteries have attracted much attention from researchers around the world.

Based on advanced theoretical algorithms, modeling, simulation, and computer technology, the reasonable design of materials, batteries, devices and batteries in the field of lithium ion batteries is gradually being realized.^13,14^ Some researchers have also used the first principle to do research about the stability on electrode materials for magnesium batteries.^15^ The calculation and experiment of large shared database connection can greatly help to narrow some of the current experimental and technical gaps, as well as predict the path-independent characteristics, and help to fundamentally understand the path-independent performance in multiple space-time scales.

Magnesium ion battery is mainly composed of three parts: compound cathode which can reversibly imbed–detach magnesium ion, metal magnesium and its alloy's negative electrode, and electrolyte.^16–18^ When discharging, the metal magnesium of the negative electrode dissolves out and forms the magnesium ion. It reaches the positive electrode through a series of processes such as dissolution, adsorption and transfer of electrolyte, and then it is embedded in the cathode materials. When charging, the magnesium ions embedded in the cathode material are detached and reach the negative electrode under the action of external electric field, and then they are deposited again to form the metal magnesium.^19–22^

At present, the studies on magnesium ion batteries at home and abroad mainly focus on cathode materials and electrolytes, which are all still in the preliminary stage. Cathode material is the key factor which restricts the development of magnesium ion battery, and some domestic and overseas scholars are trying to find and develop new cathode materials.^23–25^ MnO_2_ is a kind of electrode material with high theoretical specific capacity, low cost and easy availability, which has been widely used as battery electrode material, but its poor conductivity and stability limit the application of MnO_2_ as electrode material.^26–28^ Therefore, many researchers have improved the electrochemical performance of MnO_2_ by improving MnO_2_'s nanostructure or combining MnO_2_ with some conductive materials.^29–32^

MXene is a new type of transition metal carbide, carbonitride and nitride (which are collectively called MXene) with two-dimensional graphene-like structure. The general formula of MXene is M_*n*+1_X_*n*_T_*x*_ (*n* = 1, 2, 3), of which is an early transition metal, X is carbon or nitrogen, and T_*x*_ represents O^2−^, OH^−^, F^−^ and other surfaces functional group, MXene has been widely used in various fields.^33–35^ MXene is mainly obtained by selectively etching a fixed atomic layer in the MAX phase of the precursor. MAX phase is the general term of a class of ternary layered compounds, which have a uniform chemical formula M_*n*+1_AX_*n*_ (A is III, IV main group elements). Common chemical etchants are hydrofluoric acid or a mixture of hydrochloric acid and lithium fluoride.^36–38^ MXene is a new type of two-dimensional layered material, which has the following advantages as electrode material: (1) good electrical conductivity, which is conducive to the electron transmission; (2) large specific surface area, which can provide more storage sites; (3) layered structure, which is conducive to the rapid diffusion of electrolyte ions between layers, providing excellent rate performance; (4) high density of magnesium metal, which makes the volume specific capacity high; (5) adjustable surface chemical structure, which means different MXene can provide different working potential windows.^39–43^ So far Ti_3_C_2_T_*x*_ is the most studied MXene. It has been successfully used as electrode material for supercapacitors, sodium-ion batteries and lithium-ion batteries.^44–46^ Among them, Ti_3_C_2_T_*x*_ as electrode material for lithium ion battery is the most widely studied. Some researchers have also prepared some Ti_3_C_2_T_*x*_ matrix composites for lithium batteries and shows excellent capacity and rate performance.^47,48^ Moreover, MXene has excellent optical, mechanical and thermal stability properties, and has a wide range of applications in the fields of electromagnetic shielding, water treatment, gas, biosensor and photo electrochemistry catalyze.^49,50^

According to the advantages of MXene-a new type of material, we deposited MnO_2_ nanoparticles on the surface of MXene–Ti_3_C_2_ material with high specific surface areas and high electrical conductivity by hydro-thermal method in this article, and its electrochemical performance was tested as the cathode for magnesium batteries.

## 2. Experimental section

### 2.1 Preparation of manganese dioxide

Firstly, 145.29 mg of potassium permanganate was weighed and placed into the beaker; added 50 mL distilled water, stirred it for 30 minutes to fully dissolve. Then the solution was transferred to the hydro-thermal reactor and reacted at 150 °C for 20 h. After the reaction is over, take out the hydro-thermal reactor and cool it to room temperature. After cooling, the samples were centrifugally washed with alcohol and water alternately for 6–8 times. After that the samples were transferred to the beaker and dried in vacuum for 24 h at 60 °C, then it became the manganese dioxide.

### 2.2 Preparation of MXene–Ti_3_C_2_

Firstly, 2 g of Ti_3_AlC_2_ 2 g and lithium fluoride were respectively accurately weighed; 40 mL of hydrochloric acid (HCl, 9 M) was weighed using a measuring cylinder and was poured slowly into a beaker. Added 2 g of lithium fluoride and stirred it for 60 minutes; added 2 g of Ti_3_AlC_2_ slowly into the mixed solution; then magnetically stirred it at room temperature (25 °C) for 24 h at a speed of 200 rpm; repeated washing it ultrasonically centrifugally with deoxidized water until the pH value reaches 6; dried the sediment in vacuum at 120 °C for standby use, and this is the MXene–Ti_3_C_2_.

### 2.3 Preparation of MnO_2_/MXene–Ti_3_C_2_ composites

Firstly, 40 mg of Ti_3_C_2_T_*x*_ was weighed and placed in the beaker; added 50 mL distilled water, ultrasonically stirred it for 30 minutes; 145.29 mg of potassium permanganate with the mole ratio of 1 : 1 was weighed and fully mixed for 30 min. Then poured the stirred solution into the hydrothermal reactor, and set the temperature at 150 °C and the time at 20 h. After the reaction is over, the reaction still was taken out and cooled to room temperature. After cooling, the resulting solution was centrifuged to get the underlayer sediment; the sediment was washed with absolute ethyl alcohol and distilled water alternately for 6–8 times. The washed sample was put into the beaker and dried in a vacuum drying box at 60 °C for 24 h to obtain MnO_2_/MXene–Ti_3_C_2_ composites.

When preparing MnO_2_/MXene–Ti_3_C_2_ composites with molar ratios of 1 : 2, 1 : 1 and 2 : 1, the mass of KMnO_4_ was maintained the same, and different mass of MXene–Ti_3_C_2_ mixtures were weighed respectively for reaction, which was consistent with the above-mentioned process.

## 3. Results and discussion

### 3.1 XRD and SEM analysis

**Fig. 1:**
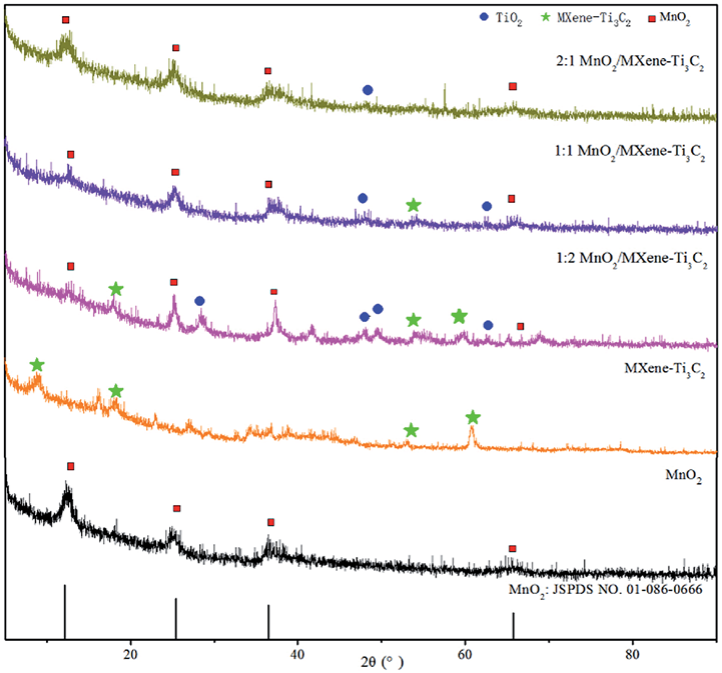
X-ray diffraction analysis results.

Fig. 1 shows the XRD spectrums of MnO_2_ and MnO_2_/MXene–Ti_3_C_2_ nanocomposites with different molar ratios. It can be seen from the figure that there are obvious diffraction peaks in MnO_2_ sample at 2*θ* of 12.77°, 24.78°, 36.55° and 66°, corresponding to (003), (006), (012) and (110) crystal planes of the δ-MnO_2_ standard card (JCPDS 01-086-0666) respectively.^51–53^ No other phase substances were detected in the spectrums, indicating that the layered δ-MnO_2_ is successfully prepared. From the XRD diffraction spectrums of MXene–Ti_3_C_2_, by comparing and analyzing the standard cards of precursor Ti_3_AlC_2_ in other papers, it can be seen that when 2*θ* was 9.0°, 16.21°, 18.32°, 34.26°, 52.88° and 60.81°, it was corresponding to the crystal planes of (002), (004), (006), (0010), (0012) and (110) respectively, these are the characteristic diffraction peaks of MXene–Ti_3_C_2_, which are consistent with other reports.^54,55^ In the XRD spectrums of MnO_2_/MXene–Ti_3_C_2_ composites with molar ratio of 1 : 2, the characteristic diffraction peaks of MnO_2_ and MXene–Ti_3_C_2_ appeared, which indicated that MnO_2_ was successfully loaded on the MXene–Ti_3_C_2_, MnO_2_/MXene–Ti_3_C_2_ composites are successfully prepared. But there is a main peak at 26.5° which illustrates that MXene–Ti_3_C_2_ is easy to be oxidized to form TiO_2_ in the hydro-thermal reaction. Moreover, the characteristic diffraction peaks of MXene–Ti_3_C_2_ were obviously weakened after adding MnO_2_. In the XRD spectrums of MnO_2_/MXene–Ti_3_C_2_ composites with molar ratio of 1 : 1 and 2 : 1, the characteristic diffraction peaks of MnO_2_ appeared, but the characteristic diffraction peaks of MXene–Ti_3_C_2_ gradually weakened and disappeared, showing that MnO_2_ nanoparticles can inhibit the recrystallization of two-dimensional MXene–Ti_3_C_2_ lamellae in MnO_2_/MXene–Ti_3_C_2_ composites, and the larger the proportion, the more obvious the effect.

**Fig. 2:**
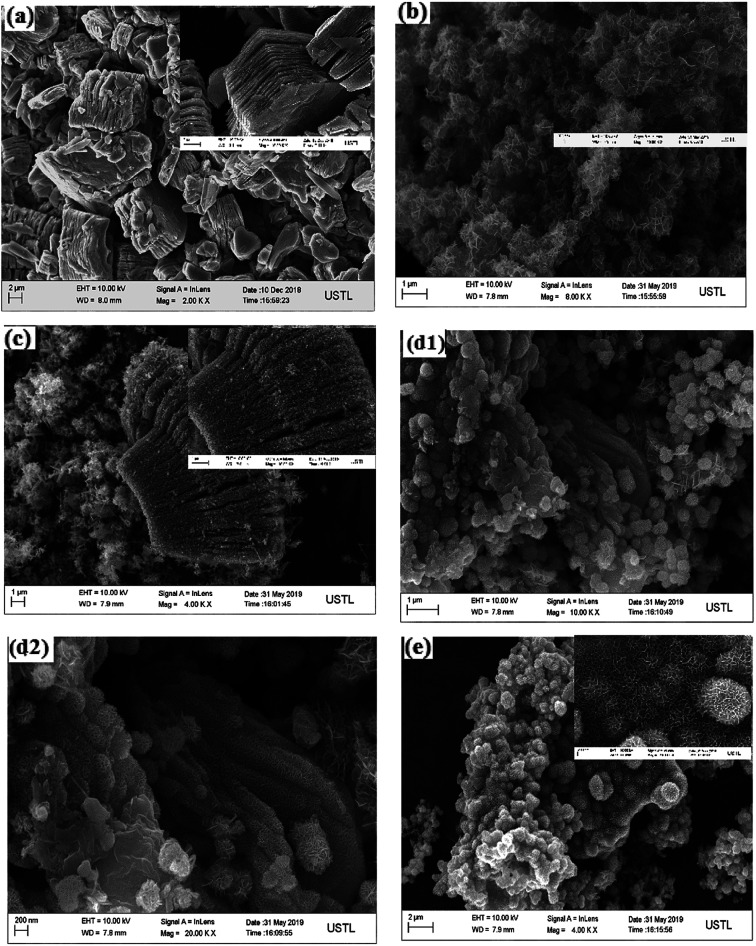
(a) SEM image of MXene–Ti_3_C_2_ material. (b) SEM image of MnO_2_ material. (c)–(e) is the MnO_2_/MXene–Ti_3_C_2_ nanocomposites with molar ratio 1 : 2, 1 : 1 and 2 : 1 in sequence.

Fig. 2 is the scanning electron microscope images of the samples at different multiples. It can be seen from the Fig. 2a that MXene–Ti_3_C_2_ has a layered structure similar to the organ, and the nanosheet of MXene–Ti_3_C_2_ are tightly bonded together, so the large specific surface area of MXene–Ti_3_C_2_ can not be fully utilized. Fig. 2b is a typical SEM image of layered MnO_2_. It can be seen that MnO_2_ is formed by overlapping and agglomeration of nanosheet layers into three-dimensional flower bract shape. The interspace of nanosheet layers is large which forms abundant pore structures.^56–59^Fig. 2c is a SEM image of MnO_2_/MXene–Ti_3_C_2_ nanocomposites with molar ratio of 1 : 2, from which we can see that MnO_2_ is evenly distributed between the nanosheet layers and the interspace of MXene–Ti_3_C_2_. At the same time, there are some irregular nanoparticles on the surface and between layers of MXene–Ti_3_C_2_, which consistent with the XRD analysis above. So MXene–Ti_3_C_2_ is easy to be oxidized to form TiO_2_ in the hydro-thermal reaction. Because MXene–Ti_3_C_2_ is obtained by removing A by etching MAX through HF, some surface functional groups will be produced during etching and some defects may occur at the edge of MXene–Ti_3_C_2_ lamellae. These defects and active sites provided by surface functional groups create conditions for the uniform distribution of MnO_2_. Fig. 2d1 and d2 is a SEM image of MnO_2_/MXene–Ti_3_C_2_ nanocomposites with molar ratio of 1 : 1 at different multiples. From Fig. 2d1, we can see that MnO_2_ nanospheres and some agglomerated TiO_2_ particles are closely accumulated on MXene–Ti_3_C_2_ lamellae, wrapping MXene–Ti_3_C_2_ sheet lamellae, and the structure of MXene–Ti_3_C_2_ can hardly be seen. As can be seen in Fig. 2d2, MXene–Ti_3_C_2_ lamellae surfaces are densely packed with spherical MnO_2_, which seriously hinders the effect of MXene–Ti_3_C_2_. Fig. 2e is a SEM image of MnO_2_/MXene–Ti_3_C_2_ nanocomposites with molar ratio of 2 : 1. In the picture we cannot see any MXene–Ti_3_C_2_ substance. There were all nano MnO_2_ with flower sphere shape. The flower spheres are tightly packed together, forming a honeycomb three-dimensional structure. In conclusion, when the molar ratio of MnO_2_ to MXene–Ti_3_C_2_ is 1 : 2, they are evenly combined. With the increase of molar ratio, the MXene–Ti_3_C_2_ substance decreases and disappears gradually. The increase of MnO_2_ inhibits the recrystallization of MXene–Ti_3_C_2_.

### 3.2 Electrochemical performance analysis

**Fig. 3:**
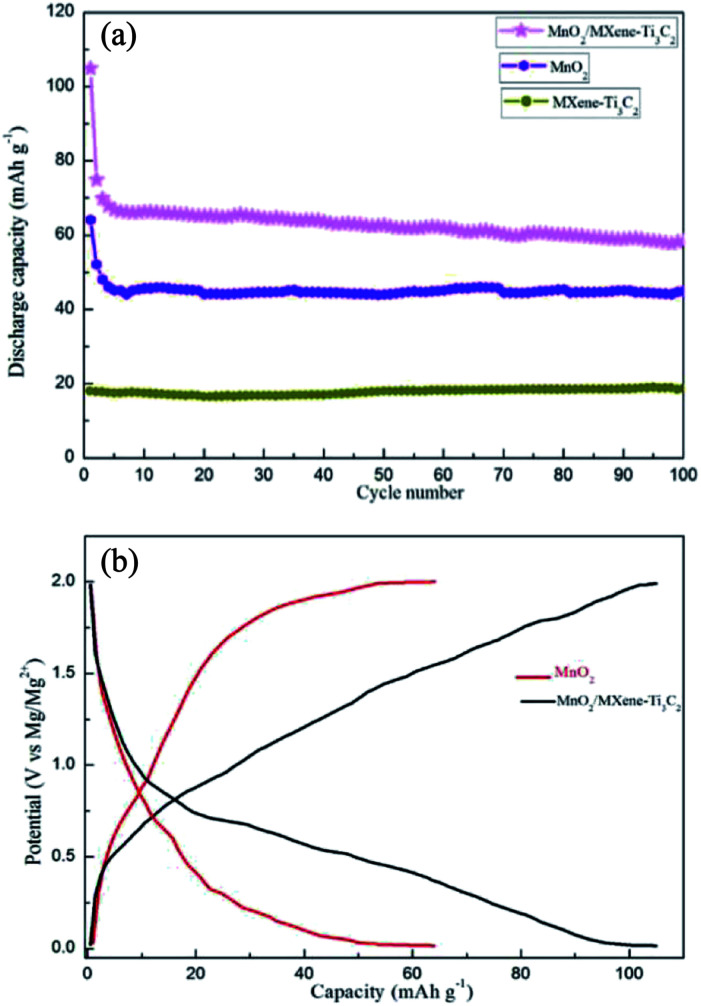
(a) Cycle performance of MXene–Ti_3_C_2_, MnO_2_ and MnO_2_/MXene–Ti_3_C_2_ composites with the molar ratio of 1 : 2. (b) Capacity–potential diagram of MnO_2_ and MnO_2_/MXene–Ti_3_C_2_.

As shown in Fig. 3a, the capacity of MXene–Ti_3_C_2_ as cathode material for magnesium batteries is 18 mA h g^−1^ at 50 mA g^−1^ current density, which is low. This is consistent with other literature reports. The first discharge capacity of MnO_2_ at 50 mA g^−1^ current density is only 64 mA h g^−1^, which is reduced to 45 mA h g^−1^ after 100 cycles. When 1 : 2 MnO_2_/MXene–Ti_3_C_2_ composite is used as cathode material of magnesium battery, the first discharge capacity is 105 mA h g^−1^, which is much higher than that of other manganese dioxide matrix composites. And after 100 cycles, the discharge capacity is 58 mA h g^−1^, the cycling performance is better than that of Mn_2_. After adding the MXene–Ti_3_C_2_, the capacity of the composite material is greatly improved, which is due to the addition of MXene–Ti_3_C_2_ which has large specific surface area and high electrical conductivity, providing more storage sites for the entry and exit of magnesium ions during the working of magnesium batteries, and shortening the transport path of magnesium ions. Fig. 3b shows the voltage–capacity curves of the two samples in the range of 0.01–2.0 V, and the average voltage is 1.0 V. The discharge curves are similar to those of capacitive storage electrode materials. As shown in Fig. 3b, we can see the MoO_2_/MXene–Ti_3_C_2_ displays that most capacity does not come from characteristic behaviors of MoO_2_ from the slope between 0.2–1.0 V for the composite. Because we added some carbon black as conductive agent in the process of assembling the electrode sheet, it is also beneficial to improve the capacity.

**Fig. 4:**
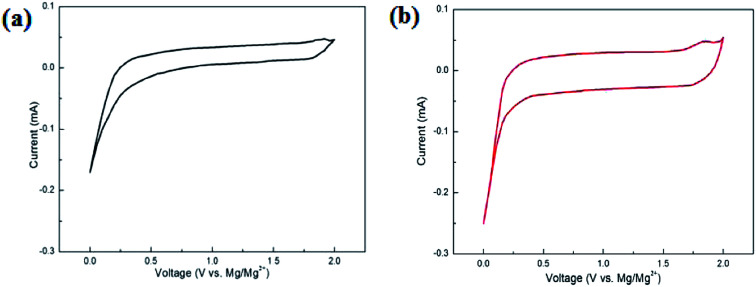
CV figure of (a) MnO_2_ and (b) MnO_2_/MXene–Ti_3_C_2_.

Fig. 4 shows the CV curves of the MnO_2_ and MnO_2_/MXene–Ti_3_C_2_ composites in the voltage range of 0.01–2.0 V. Compared with MnO_2_, MnO_2_/MXene–Ti_3_C_2_ composite material has obvious anode and cathode current response, large CV area and obvious capacity. The CV curve shows a sharp peak when it's lower than 0.2 V, which may be due to Mg^2+^ embedded at the active site of the electrode with high activation energy. This is consistent with the relatively flat discharge curve in the low voltage region of 0.01–0.2 V shown in Fig. 3b. However, after the battery is stabilized, the curve of charge and discharge voltage between 0.01–2.0 V no longer shows a clear charge and discharge platform, which is similar to the charge and discharge curve of capacitor storage, indicating that the main energy storage mechanism of MnO_2_/MXene–Ti_3_C_2_ sample is embedded pseudocapacitance, and the coulombic efficiency of the electrode material is close to 100%. MnO_2_/MXene–Ti_3_C_2_ sample has good cycle performance.

**Fig. 5:**
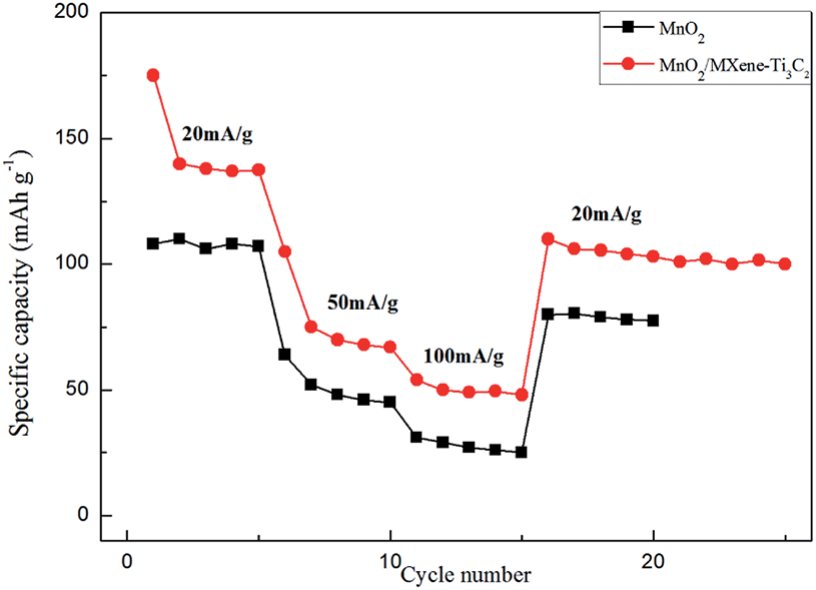
Rate performance of MnO_2_ and MnO_2_/MXene–Ti_3_C_2_.

MnO_2_/MXene–Ti_3_C_2_ composite electrodes also show excellent rate performance. As shown in Fig. 5, at high current densities of 100 mA g^−1^, the capacities are 54 mA h g^−1^, which are much higher than those of MnO_2_. In the first ten circles, there is some attenuation of capacity, which is due to the strong electrostatic interaction between Mg^2+^ and electrode materials, and Mg^2+^ is easy to be captured by some active sites. At a high current density of 100 mA g^−1^, the battery still has a stable capacity of 54 mA h g^−1^ and still has a high capacity at a wide range of current densities, indicating that MnO_2_/MXene–Ti_3_C_2_ is a very nice high rate cathode material for magnesium batteries.

## 4. Conclusion

MnO_2_ and MnO_2_/MXene–Ti_3_C_2_ composites with different molar ratios are successfully prepared by one-step hydrothermal method in this paper. When the molar ratio was 1 : 2, MnO_2_ evenly adhered on and between MXene–Ti_3_C_2_ lamella, and the composite effect was the best, which was conducive to the imbedding and separation of magnesium ions. When 1 : 2 MnO_2_/MXene–Ti_3_C_2_ composite is used as cathode material of magnesium battery, the main energy storage mechanism is embedded pseudo capacitance. The first discharge capacity at 50 mA g^−1^ current density is 105 mA h g^−1^, which is much higher than that of MnO_2_. Even at a high current density of 500 mA g^−1^, the MnO_2_/MXene–Ti_3_C_2_ composite battery still has a stable capacity of 21 mA h g^−1^ and good rate performance. At the same time, MXene–Ti_3_C_2_ substrate material with high conductivity is very valuable for the study of new electrode materials.

## Funding

This work was supported by the Magnesium Industry Collaborative Innovation Center of University of Science and Technology Liaoning (USTLXT201801), Liaoning Natural fund project (2019-ZD-0027) and National Science and Technology Support Plan (2014BAB02B01).

## Conflicts of interest

There are no conflicts to declare.

## Supplementary Material
